# Pediatric non alcoholic fatty liver disease: more on novel treatment targets

**DOI:** 10.1186/1471-2431-13-109

**Published:** 2013-07-19

**Authors:** Pietro Vajro, Giulia Paolella, Marco Poeta, Cristina Pizza, Maria Sangermano, Grazia Massa

**Affiliations:** 1 Department of Medicine and Surgery, University of Salerno, Via S. Allende, 84081, Baronissi -Salerno, Italy

**Keywords:** Non alcoholic fatty liver disease, Children, Gut-liver axis, Microbiota, Probiotics, Therapy

## Abstract

The mainstay treatment of non alcoholic fatty liver disease (NAFLD) based on weight loss and/or lifestyle changes is most often unsuccessful at all ages, thus requiring the implementation of pharmacological strategies. Targeting insulin resistance and oxidative stress has recently proven unsatisfactory. Among a number of proposed innovative approaches targeting novel pathomechanisms, probiotics appear an interesting and reasonable option acting on gut-liver axis malfunction through the modulation of diet-driven, obesogenic, and inflammatory intestinal microbiota.

A combined multiple pharmacological therapy directed simultaneously towards novel and old pathomechanisms (including, e.g., insulin resistance, oxidative stress, gut-liver axis, apoptosis) along with lifestyle interventions however might be necessary both in adult and pediatric NAFLD therapy.

## Commentary

The available treatment options for NAFLD at all ages are currently still far from being satisfactory. Therefore we read with much interest the article by Giorgio et al. which appeared most recently in the Journal [1]. The Authors gave pediatricians a thorough update on several aspects of NAFLD pathomechanisms, which were reviewed also under the light of potential treatments targets. Dr Giorgio et al. should be congratulated in particular for the attention paid also to the intestinal microbiota and the gut-liver axis. These issues in fact only recently emerged as additional, possibly pivotal, protagonists in the busy scenario of NAFLD pathogenesis not only in animal models [2] but also in humans, including childhood [3]. This now begins to appear paramount due to intestinal microbiota’s susceptibility of being modulated by a number of agents (including pre-, pro-, and syn-biotics) [4].

Since the 2007 Cochrane meta-analysis [5], which could neither suggest nor refute the use of probiotics as therapeutic option for patients with NAFLD and non-alcoholic steatohepatitis (NASH), most recently at least two double blind, controlled, pilot RCTs (both with probiotics as the sole treatment) became available in the literature [6,7]. Both studies showed encouraging preliminary good results upon aminotransferases activity in adults [6] and, even more, in pediatric population where also a number of markers of intestinal dysbiosis and/or gut-liver axis malfunction appeared to be modulated by probiotic treatment [7]. Hence, these results, altogether, appear to nicely fit the intense network existing between gut and liver and probiotics influence [3,4] which also Giorgio et al. mentioned in their study [1]. Moreover they may add further strength to the Authors’ view that large, well conducted, controlled studies with probiotics or other therapeutic tools able to interact with the gut microbiota, alone and/or together with other therapeutic measures (i.e. “multi-target therapy” avoiding the monotherapeutic direction approach) [4,8], appear fully justified also in pediatric NAFLD.

At the same time, for future NAFLD therapeutic studies, one should underline the importance of warning on the strict monitoring patients’ anthropometric changes to avoid under/over-estimation of the effects of the therapies under consideration. Unpredictable concurrent individual lifestyle changes in fact represent a relevant confounding factor which has hitherto flawed a number of medium and long-term studies in both pediatric and adult obese populations [4]. Accordingly, also the categorical need for the assessment of “biopsy proven” basal and/or follow-up histological liver damage should be cautiously reconsidered. In addition to the unpredictability of sampling error potentials, one should bear in mind that liver biopsy results may in fact represent only a transient & mutable snapshot during the extremely irregular lifestyle- and diet-driven hepatic histological changes, especially in the pediatric age.

## Abbreviations

(NAFLD): Non alcoholic fatty liver disease; (NASH): Non-alcoholic steatohepatitis.

## Competing interests

The authors declare that there is no competing interest regarding the material discussed in the manuscript.

## Authors’ contributions

PV and GP –prepared the first draft of manuscript. MP, CP, MS, and GM– prepared bibliographical background. PV – was the guarantor of manuscript, supervised, revised the manuscript. All authors read and approved the final manuscript.

## Pre-publication history

The pre-publication history for this paper can be accessed here:

http://www.biomedcentral.com/1471-2431/13/109/prepub
